# Progress on the Maternal Mortality Ratio Reduction in Wuhan, China in 2001–2012

**DOI:** 10.1371/journal.pone.0089510

**Published:** 2014-02-21

**Authors:** Shaoping Yang, Bin Zhang, Jinzhu Zhao, Jing Wang, Louise Flick, Zhengmin Qian, Dan Zhang, Hui Mei

**Affiliations:** 1 Department of Primary Guidance, Wuhan Women and Children Health Care Center, Wuhan, Hubei Province, China; 2 College of Public Health and Social Justice, St. Louis University, St. Louis, Missouri, United States of America; Université de Montréal, Canada

## Abstract

**Background:**

Most maternal deaths occur in developing countries and most maternal deaths are avoidable. China has made a great effort to reduce MMR by three quarters to meet the fifth Millennium Development Goal (MDG5).

**Methods:**

This retrospective study reviewed and analyzed maternal death data in Wuhan from 2001 to 2012. Joinpoint regression and multivariate Poisson regression was conducted using the log-linear model to measure the association of the number of maternal deaths with time, cause of death, where the death occurred, and cognitive factors including knowledge, attitude, resource, and management stratified.

**Results:**

The MMR declined from 33.41 per 100,000 live births in 2001 to 10.63 per 100,000 live births in 2012, with a total decline of 68.18% and an average annual decline of 9.89%. From 2001–2012, the four major causes of maternal death were obstetric hemorrhage (35.16%), pregnancy complications (28.57%), amniotic fluid embolism (16.48%) and gestational hypertension (8.79%). Multivariate Poisson regression showed on average the MMR decreased by.17% each year from 2001–2006 and stayed stagnant since 2007–2012.

**Conclusions:**

With the reduction in MMR in obstetric death (e.g. obstetric hemorrhage), there had been a remarkable reduction in MMR in Wuhan in 2001–2012, which may be due to (1) the improvement in the obstetric quality of perinatal care service on prevention and treatment of obstetric hemorrhage and emergency care skills, and (2) the improvement in the maternal health management and quality of prenatal care. Interventions to further reduce the MMR include several efforts such as the following: (1) designing community-based interventions, (2) providing subsidies to rural women and/hospitals for hospital delivery, (3) screening for pregnancy complications, and (4) establishing an emergency rescue system for critically ill pregnant women.

## Introduction

Reducing maternal mortality is a continuing global priority. According to the World Health Organization (WHO), approximately 800 women die every day from preventable causes related to pregnancy and childbirth. Over 99% of these deaths occur in developing countries [1]. The maternal mortality ratio (MMR) in developing countries is 240 per 100,000 births versus 16 per 100,000 in developed countries in 2010 [2]. The fifth Millennium Development Goal (MDG5) aimed to reduce maternal mortality by 75% in 1990–2015 [3]. The global MMR was 210 per 100,000 live births in 2010 compared with 400 in 1990, with an approximate total decline of 50%. However, the global MMR declined by only 3.1% per year, lower than 5.5% set by MDG5 [1], [4], [5], and therefore improving maternal health remains a key global priority.

Many developing countries are making encouraging progress towards reducing their MMR. China, as a developing country with a population over 1.3 billion, has placed great emphasis on maternal and child health, and has made considerable progress in reducing the MMR in the past 60 years, although wide regional variations in maternal mortality levels exist in China. China endorsed the MDG5 in 2000 and promised to reduce the MMR to 22 per 100,000 live births by 2015 [5]. The MMR in China has dropped from 95 per 100,000 live births in 1990 to 30 in 2010 [6], with a total decline of 68.4% and an annual decline of 5.9% per year. China is on track nationally for most of the MDGs [2].

In China causes of maternal death are related to biomedical, reproductive, health service, socioeconomic and cultural factors [7]–[10]. Biomedical factors include direct and indirect obstetric causes. The three major direct causes of maternal death in China are postpartum hemorrhage (28.0%), gestational hypertension (12.3%), and heart disease (10.9%) [6], in contrast to developed countries, preventable obstetric causes are still the main cause for maternal mortality in China. Reproductive causes include both reproductive age and the number of births per woman during her reproductive years. The MMR is defined as the annual number of maternal deaths divided by the total number of live births and then multiplied by 100,000. Thus, the MMR reflects both the risk of maternal death per pregnancy or per birth. Lifetime risk is the probability that a woman will die from complications of pregnancy and childbirth over her lifetime; it takes into account both the MMR and the mother’s total fertility (probable number of births per woman during her reproductive years). Thus in a high-fertility setting a woman faces the risk of maternal death multiple times, and her lifetime risk of death will be higher than in a low-fertility setting. The risk is highest for adolescent girls under 15 years of age and women above 35 years of age [11], [12]. Although China has upheld a strict one-child policy since 1979 and strengthened the health care provided to women pregnant over 35 years of age, the lifetime risk of maternal death in China is 1 in 1700, versus 1 in 3800 in developed countries [2].

According to WHO, the high number of maternal deaths in some areas of the world reflects inequities in access to health services and highlights the gap between rich and poor. In China, there are large disparities between people with high and low income, between people living in rural and urban areas, and between migrants and permanent residents [7], [13], [14]. But aggressive efforts to improve quality and availability of prenatal and delivery services have improved conditions. The Chinese Government has implemented programs to reduce disparities in access to prenatal and perinatal services and to make more affordable and effective treatment available through developing training programs for health workers, and implementing healthcare policies and monitoring systems for pregnant women.

This paper offers a case study of one region’s response to the Chinese government’s effort to reduce MMR. Wuhan, the largest and most prosperous city of central China with 9.79 million residents has made good progress with a reduction in MMR from 33.41 per 100,000 live births in 2001 to 10.63 in 2012, with a total decline of almost 68.18% and an annual decline of 9.89%. Collecting and analyzing such maternal mortality data over time allows evaluation of progress and planning for rational allotment of health resources. The goal of the current study is to describe the progress that Wuhan has made since 2000 in improving maternal health care and reducing the MMR, and the efforts that brought about this change.

## Methods

### Subject Selection

Our source population was the cohort of pregnant women who resided in Wuhan for at least one year. According to WHO, maternal death is defined as the death of a woman while pregnant or within 42 days of termination of pregnancy, irrespective of the duration and site of the pregnancy, from any cause related to or aggravated by the pregnancy or its management but not from accidental or incidental causes. A total of 91 maternal deaths occurred during the study period.

Reporting of maternal deaths was done via two systems: the maternal and child health information monitoring system and a three-tier reporting system of maternal and child care at the city, district/county, and community levels in Wuhan. Information about maternal deaths was contained in the subject’s report card which records when, where, and how each death occurred, death questionnaire, investigation report, medical record, perinatal care guide, or municipal assessment, described as follows. First, all pregnancies were confirmed by the doctors using pregnancy tests or B-mode ultrasound examination, and registered in the maternal and child health information monitoring system. Health care service providers then reported maternal deaths to maternal and child health care institutions at the district level which serves the region using hospital records or through home visits for maternal deaths. All information was reported to maternal and child health care institutions at the city level (Wuhan Women and Children Health Care Center), and then the city level summarized and evaluated for validity, reliability, and accuracy quarterly. The International Statistical Classification of Diseases and Related Health Problems 10th Revision (ICD-10) diagnostic codes by the World Health Organization (WHO) were used to classify diseases and other health problems by the evaluation experts at city and district levels. Discrepancies between city and district evaluation reports would then be identified and corrected according to the city level assessment results.

A retrospective study was carried out to review and analyze the data on maternal death in Wuhan from 2001 to 2012. The Twelve Lattice Law of WHO was adopted to determine the factors for maternal death, including individuals/families, health care institutions, and other institutions. According to the evaluation standard of maternal death review of Ministry of Health of the People’s Republic of China, causes of death were categorized as: 1) avoidable, 2) avoidable when the needs that allow to avoid maternal death such as health care, individual living necessities, medical facilities were provided, named as conditionally avoidable, and 3) unavoidable. The conditionally avoidable category was dropped in 2006.

This study was based on the official registration in the system and approved by the Health Department of Hubei Province and the Institutional Review Board at Wuhan Women and Children Health Care Center. Written informed consent forms were obtained from all family members of death pregnant and puerperal women involved in this study.

### Statistical Analysis

Descriptive statistics, such as frequencies and percentages of baseline variables were calculated. Pearson correlation coefficients were calculated to measure the associations between two variables. Significance was declared when *p*<0.05. We considered joinpoint regression to identify the number of join points and tested whether a multi-segmented line was a significantly better fit than a straight or less-segmented line. This involved fitting a series of joined straight lines on a log scale to the trends in number of maternal death. Line segments that were joined at points were called joinpoints. Accordingly, joinpoint regression and multivariate Poisson regression was conducted using the log-linear model to measure the association of the number of maternal deaths with time from the baseline (2001) to the end of the study period (2012), cause of death (obstetric hemorrhage, pregnancy complications, amniotic fluid embolism, gestational hypertension, anesthetic accident, ectopic pregnancy, and other), where the death occurred (provincial/municipal hospital, county hospital, township hospital, private clinic, home, transport vehicle), and cognitive factors including knowledge, attitude, resource, and management stratified by 3 groups of subjects: individuals/families, health care institutions, and other institutions. All statistical analyses were performed using SAS 9.3 (SAS Institute, Cary NC).

## Results

### The Trends of MMR in Wuhan

**Figure 1:**
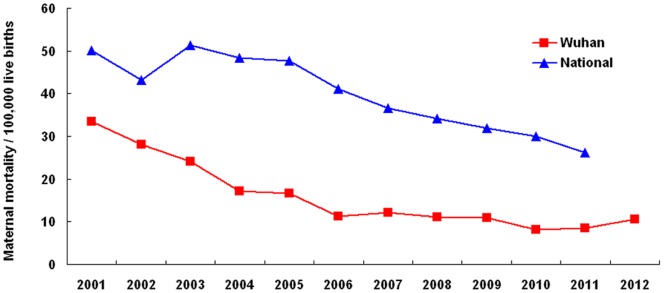
The maternal mortality ratios in 2001–2012 for Wuhan and all cities in China combined.

The MMR in Wuhan appeared lower than the national average MMR from 2003–2012 (Figure 1) and all pregnant women in Wuhan have given birth in a hospital since 2009 (Table 1). From 2001–2012, the numbers of live births in Wuhan increased by 93.40% and the MMR decreased by 68.18%, with an average annual decrease of 9.89%. A rapid average annual decrease of 19.42% was seen in 2001–2006 and after 2007 the decrease rate has slowed, with an average annual decrease of 2.72%. The Pearson correlation coefficient between the number of maternal deaths and time was estimated to be −.98 with a p value of.0006 from 2001–2006, i.e., the number of deaths was moderately, negatively related to time during that time period, and there was no significant correlation between the number of maternal deaths and time after 2006. Figure 1 displays two straight lines connected together at the “join points”. Each joinpoint denoted a statistically significant (p value <.05) change in trend (Table 2). The test of significance used a Monte Carlo Permutation method (i.e., it found “the best fit” line for each segment). According to Figure 1, we chose a minimum of zero joinpoints (one line segment) and a maximum of 1 joinpoint (two line segments) to test whether 1 joinpoint was statistically significant and must be added to the model.

**Table 1:** Frequency of live births and maternal deaths, MMR, and percent of pregnant women who gave birth in a hospital in Wuhan in 2001–2012.

Year	Number of live births	Number of maternaldeaths	MMR (per 100,000 live births)	Hospital delivery (%)
2001	38910	13	33.41	95.45
2002	39270	11	28.01	97.15
2003	37443	9	24.04	98.28
2004	40618	7	17.23	99.47
2005	41955	7	16.68	99.68
2006	44053	5	11.35	99.81
2007	49165	6	12.20	99.97
2008	54125	6	11.09	99.97
2009	64325	7	10.88	100
2010	74063	6	8.10	100
2011	70365	6	8.53	100
2012	75252	8	10.63	100

**Table 2:** Maternal mortality rate.

JoinPoint Regression (2001–2011)	
Trend 1 Years APC** (p value)	Trend 2 Years APC** (p value)
−.21∧ (.0003)	−.05 (.13)

Average annual maternal mortality rate** (2001–2011) and trends (JoinPoint Analyses for 2001–2011) pregnant women who gave birth in Wuhan.

*Average annual maternal mortality rate are per 100,000.

**APC = Annual percent change calculated by using joinpoint regression analysis.

∧APC is significantly different from zero (two-side p<.05).

Notes: Joinpoint analysis allowed for up to two joinpoints and are based on rate per 100,000.

**Figure 2:**
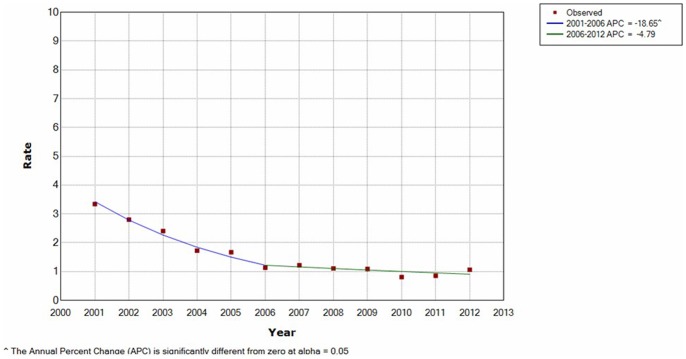
Maternal mortality rate by joinpoint regression.

According to the results of the joinpoint analysis, it was determined that the maternal direct death incidence rates decreased dramatically by.17% per year until 2006, and then stabilized. The resulting curves fitted by joinpoint regression was given in Figure 2.

**Figure 3:**
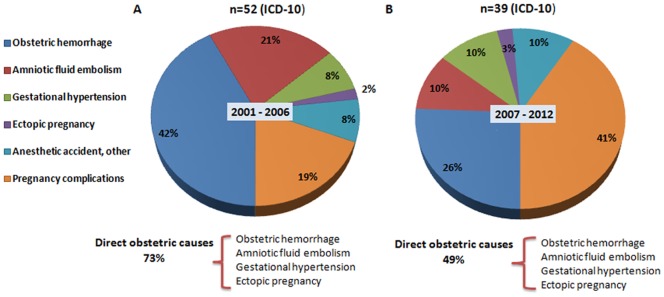
Relative importance of 6 different direct causes of maternal death in Wuhan during 2 periods between 2001 and 2012. A: Obstetric haemorrhage, B: Pregnancy complications, C: Amniotic fluid embolism, D: Gestational hypertension, E: Anesthetic accident, Ectopic pregnancy, Other.

We then divided the 12 time periods (2001–2012) into two intervals, 2001–2006 and 2007–2012, and used this binary time variable as a covariate in the subsequent analyses. Figure 3 displays the pie chart of the frequency of each type of causes of maternal death [15]. The percentage of mortality due to obstetric hemorrhage and amniotic fluid embolism decreasesd greatly in the two periods wherease the percentage of mortality due to the other three causes increased, with the increase due to pregnancy complications being substantial (from 19% to 41%). Obstetric hemorrhage was the leading cause of maternal death in the first period (42%) wherease pregnancy complications was the leading cause of maternal death in the second period (41%). The percentage of mortality due to direct causes of maternal death (obstetric hemorrhage, amniotic fluid embolism, gestational hypertension, and ectopic pregnancy) decreased from 73% to 49% in the two periods. In other words, the percentage of mortality due to indirect causes of maternal death (Anesthetic accident and pregnancy complications) increased from 27% to 51% in the two periods.

### Maternal Deaths Due to Direct and Indirect Obstetric Causes

Table 3 shows the percent of maternal deaths due to direct causes had decreased, whereas the percent of maternal deaths due to indirect causes had increased in Wuhan in 2001–2012.

**Table 3:** The number and proportion (%) of maternal deaths (the first row) and the MMR per 100,000 live births by cause (the second row) in Wuhan between 2001 and 2012.

Year	Obstetric hemorrhage	Pregnancy complications	Amniotic fluid embolism	Gestational hypertension	Anesthetic accident	Ectopic pregnancy	Other
2001	9 (69.2)	2 (15.4)	1 (7.7)	1 (7.7)	0 (0)	0 (0)	0 (0)
	23.13	5.14	2.57	2.57	0	0	0
2002	4 (36.4)	3 (27.3)	2 (18.2)	1 (9.1)	0 (0)	0 (0)	1 (9.1)
	10.19	7.64	5.10	2.55	0	0	2.55
2003	3 (33.3)	1 (11.1)	3 (33.3)	1 (11.1)	0 (0)	0 (0)	1 (11.1)
	8.01	2.67	8.01	2.67	0	0	2.67
2004	1 (14.3)	1 (14.3)	3 (42.9)	0 (0)	1 (14.3)	0 (0)	1 (14.3)
	2.46	2.46	7.39	0	2.46	0	2.46
2005	3 (42.9)	1 (14.3)	1 (14.3)	1 (14.3)	0 (0)	1 (14.3)	0 (0)
	7.15	2.38	2.38	2.38	0	2.38	0
2006	2 (40.0)	2 (40.0)	1 (20.0)	0 (0)	0 (0)	0 (0)	0 (0)
	4.54	4.54	2.27	0	0	0	0
2007	1 (16.7)	2 (33.3)	1 (16.7)	1 (16.7)	0 (0)	1 (16.7)	0 (0)
	2.03	4.07	2.03	2.03	0	2.03	0
2008	1 (16.7)	2 (33.3)	1 (16.7)	1 (16.7)	0 (0)	0 (0)	1 (16.7)
	1.85	3.70	1.85	1.85	0	0	1.85
2009	3 (42.9)	2 (28.6)	1 (14.3)	1 (14.3)	0 (0)	0 (0)	0 (0)
	4.66	3.11	1.55	1.55	0	0	0
2010	2 (33.3)	3 (50.0)	1 (16.7)	0 (0)	0 (0)	0 (0)	0 (0)
	2.70	4.05	1.35	0	0	0	0
2011	1 (16.7)	4 (66.6)	0 (0)	0 (0)	1 (16.7)	0 (0)	0 (0)
	1.42	5.68	0	0	1.42	0	0
2012	2 (25.0)	3 (37.5)	0 (0)	1 (12.5)	0 (0)	0 (0)	2 (25.0)
	2.66	3.99	0	1.33	0	0	2.66

The percentage of direct obstetric deaths due to obstetric hemorrhage, gestational hypertension, amniotic fluid embolism and ectopic pregnancy was 84.6% in 2001 and dropped to 37.5% in 2012, and the percentage of direct obstetric deaths was 62.64%. For direct-causes, the MMR decreased from 28.27 to 3.99 per 100,000 live births. Obstetric hemorrhage was the main cause of direct obstetric maternal deaths, causing a 54.24% of direct obstetric maternal deaths.

The percent of maternal deaths due to indirect causes increased from 15.4 in 2001 to 37.5 per 100,000 live births in 2012. Twenty-six maternal deaths were caused by the pregnancies complicated by disease, the leading indirect cause of indirect maternal death in 2001–2012 in Wuhan, with 8 due to heart diseases, 6 to acute severe pancreatitis, 5 to hepatitis or liver and kidney insufficiency, 3 to pneumonia, 2 to server influenza A (H1N1), 1 to pulmonary tuberculosis and 1 to Thrombocytopenia.

Since maternal deaths are considered as the count data, a Poisson regression model was specified with the year and direct cause of death being considered as covariate (denoted by the letter of C). Normality test using the Shapiro-Wilk method also suggested the death data did not have a normal distribution (test statistic = 0.83 and p value <0.001). Because of the nature of the Poisson distribution, by definition a log transformation of the data is employed to form the following log-linear model:
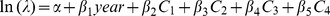
where C_1_, C_2_, C_3_ and C_4_ are the first 4 causes in Table 4 respectively, and the last three categories in Table 4 were combined into the reference category, and therefore β_2_ is interpreted as the mean number of deaths due to obstetric hemorrhage (the non-reference category) that was higher/lower than the reference category, controlling for time, and β_3_, β_4_, β_5_ were interpreted similarly. Poisson regression showed the estimates of β’s were 1.07(Chi-square = 9.3, p value = .002), .86(Wald Chi-square = 5.7, p value = .02), .31(Wald Chi-square = .6, p value = .44) and −.32 (Wald Chi-square = .5, p value = .49), i.e., controlling for time, the average numbers of death caused by obstetric hemorrhage and pregnancy complications were 2.9 and 2.4 times, respectively, the average numbers of death due to other causes (anesthetic accident, ectopic pregnancy, etc).

**Table 4:** Frequency and percent of maternal deaths in provincial/municipal hospitals, county hospitals township hospitals, private clinics, homes and 2001–2012.

Year	Provincial/municipalhospital n(%)	County hospital n(%)	Townshiphospital n(%)	Private clinicn(%)	Home n(%)	Transport vehicle n(%)
2001	9(69.2)	3(23.1)	0(0)	0(0)	0(0)	1(7.69)
2002	5(45.5)	3(27.3)	1(9.09)	0(0)	1(9.09)	1(9.09)
2003	4(44.4)	2(22.3)	0(0)	0(0)	1(11.1)	2(22.2)
2004	6(85.7)	0(0)	0(0)	0(0)	0(0)	1(14.3)
2005	1(14.3)	3(42.9)	3(42.86)	0(0)	0(0)	0(0)
2006	1(20.0)	2(40.0)	0(0)	1(20.0)	1(20.0)	0(0)
2007	2(33.3)	3(50.0)	1(16.7)	0(0)	0(0)	0(0)
2008	4(66.7)	1(16.7)	0(0)	0(0)	1(16.7)	0(0)
2009	7(100.0)	0(0)	0(0)	0(0)	0(0)	0(0)
2010	6(100.0)	0(0)	0(0)	0(0)	0(0)	0(0)
2011	6(100.0)	0(0)	0(0)	0(0)	0(0)	0(0)
2012	7(87.5)	0(0)	0(0)	0(0)	1(12.5)	0(0)

### Site of Maternal Death

About 82% maternal deaths were reported by provincial/municipal/county hospitals in 2001–2012. No maternal death occurred during delivery at home or at private clinics from 2009 to 2011 (Table 4).

We used the similar log-linear model to the above to characterize the association between the number of maternal deaths and time and where the death occurred. We combined the last 4 categories in Table 3 into the reference category. Poisson regression showed the estimates of the β’s were 1.17 (Wald Chi-square = 18.81, p value <.001) and −.06 (Wald Chi-square = .03, p value = .87), i.e., controlling for time, the average number of deaths that occurred in provincial/municipal hospitals was 3.2 times the average number of deaths occurred in other places (township hospital, private clinic, home, or transport vehicle).

### Evaluation of Maternal Death

The total number of avoidable and conditionally avoidable deaths was 68, accounting for 74.73% of the number of all maternal deaths, which were caused by the lack of knowledge or skills, problems/issues about the resource and patient management system, or poor attitudes on the part of health care providers (Table 4). The number of unavoidable maternal deaths was 23 (25.27%). The percent of avoidable and conditionally avoidable decreased from 84.60% in 2001 to 33.33% in 2011. The percent of unavoidable deaths increased from 15.40% to 66.67%.

We divided the study subjects into 3 groups, including individuals/families, health care institutions, and other institutions by knowledge and attitude (Table 5). We used the log-linear model to characterize the association between the number of maternal deaths and time, group and cognitive factor. The estimate for the slope of time is −.5 (p value = .03). The average numbers of deaths due to the lack of knowledge and attitude were 2 (Wald Chi-square = 7.3, p value = .007) and 1.6 (Wald Chi-square = 4.6, p value = .03) times, respectively, the average numbers of deaths due to the lack of management. The average numbers of deaths among individuals/families and health care institutions were 2 (Wald Chi-square = 7.3, p value = .007) and 2.26 (Chi-square = 9.4, p value = .002) times, respectively, the average numbers of deaths among other institutions.

**Table 5:** Maternal mortality due to knowledge, attitude, resource and management.

Items	2001	2002	2003	2004	2005	2006	2007	2008	2009	2010	2011	2012
Individuals/families												
Knowledge	2	2	2	1	1	1	2	1	1	0	0	2
Attitude	4	3	2	2	2	0	0	1	1	1	0	1
Health care institutions												
Knowledge	2	1	2	2	2	1	2	2	1	1	1	2
Attitude	2	0	1	0	1	1	0	0	1	0	0	0
Resource	0	1	1	1	0	0	0	1	1	0	1	1
Management	0	0	0	0	0	1	0	0	0	1	0	0
Other institutions												
Management	1	1	0	0	0	0	0	0	0	0	0	0

## Discussion

This study showed an overall downward trend in MMR in Wuhan in 2001–2012, with an annual average rate of decline of 19.42% in 2001–2006, and 2.72% in 2007–2012, showing a leveling off of the trend. Since 2006, the MMR in Wuhan has been lower than the 16 per 100,000 live births reported by WHO for the average MMR in developed countries as a group [2]. While Wuhan’s rate of decline has slowed, it is still higher than the annual average rate of decline of 1.20% of all cities in China in 2001–2010 [6], the 1.32% decline in Shanghai in 2000–2009 [16], and the 2.48% decline in Beijing from 2001–2010 [17], and such a great reduction in MMR in Wuhan occurred in the context of China’s national priorities and efforts.

Various strategies have been implemented in China over the last 60 years to improve maternal and child health care in four stages [18]. The first stage was strengthening neonatal nursing and midwifery, prevention of puerperal fever, and popularization of using sterile procedures for delivery. Midwives were prohibited from delivering babies on the ground or sand, using non-sterile packs, leaving perineal tears without repair and umbilical cords unsterilized. They were also required to attend a midwifery training program to learn and practice skills such as using sterile technique. During this stage (1950–1960) the MMR was about 500 per 100,000 live births. The second stage occurred in 1960–1980 and began with implementing maternal care management, delivering rural midwife training programs, building maternity units in rural areas, and screening for high-risk pregnancies. The problems faced at this stage were the slow popularization of using sterile procedures for delivery, the lack of advanced midwifery training programs, and a large number of maternal deaths from postpartum hemorrhage and hypertensive disorders of pregnancy. The MMR was between 100 and 500 per 100,000 live births during this stage. The third stage occurred following China’s major economic growth in 1980’s when the government increased investment in healthcare to improve the overall health of the Chinese people. The third stage began to establish maternal health care systems, promote hospital births, and start maternal mortality registration. The overall MMR was about 100 per 100,000 live births in 1980–2000. Rural areas continued to have higher MMRs than urban areas, and central and western China had a higher MMR than eastern China. The fourth stage began in 2000 following the implementation of the Chinese government’s Reduce Maternal Mortality project and a project to eliminate neonatal tetanus in 378 counties of 12 provinces/autonomous regions/municipalities in central and western China [19]. The project aimed to reduce the cost of health care for labor and birth for poor families, establish an emergency obstetric service (called Green Channel) and an emergency aid center, develop training programs for obstetrics and gynecology personnel and pediatric staff, and develop a high-quality healthcare system, reduce charges for prenatal, delivery, and postnatal care, and provide health education. Political will and commitment to improving maternal health played a key role in reducing maternal mortality [20] and resolving problems with personnel management and implementation of health education messages. This stage continued to see an increased investment in healthcare by the Chinese government. Implementation of this project led to a significant MMR reduction [19], [21], and by the end of 2006 the MMR had fallen by more than 50% in the autonomous regions of Guangxi and Ningxia, 40%–50% in Shanxi, Anhui, Henan, Hubei and Xinjiang, 30%–40% in Jilin, Hunan, Hainan, Chongqing, Sichuan and Gansu, and 20% and 10% in Tibet and Yunnan, respectively. The overall MMR in China dropped from 76 per 100,000 live births in 2001 to 49 in 2006, with a total decline of 35.8%, and an annual decline of 8.4% [19], [22], [23].

Political will and commitment played a key role in reducing maternal deaths nationally [19] and in Wuhan. Wuhan’s government has led efforts to improve maternal mortality through prohibiting illegal delivery practices, reinforcing quality maternal management practices, strengthening, monitoring and managing high-risk pregnancies, providing maternal health consulting services, creating strategies for improving the knowledge, attitude, and skill of health professionals, establishing an obstetric emergency service, and conducting periodic maternal mortality reviews and utilizing health system reviews to enhance accountability for preventable maternal mortality.

In addition the Wuhan government has made great efforts to take maternal mortality prevention measures, including but not limited to the following. Each completed maternal death reporting form is shared with a maternal death review committee. If a maternal death occurred in a health care institution which provided midwifery services, then the institution must report and investigate. If the collaborative group from the committee determines the health care institution had primary responsibility then the quality of obstetric will be deemed substandard and the regulation authorities will instruct the health care institution to make corrections. For those institutions with two avoidable maternal deaths occurring in two years their midwifery services will be terminated.

Although the number of direct obstetric deaths decreased in Wuhan in 2001–2012, direct obstetric causes were still the major causes of maternal deaths, resulting in 62.64% of maternal deaths, and post-partum hemorrhage was the leading cause of maternal deaths, causing nearly 50% of maternal deaths in Wuhan. The low-cost baby delivery services provided in rural maternity hospitals had yielded an important increase in hospital delivery in rural Wuhan and a decrease in maternal mortality resulting from obstetric hemorrhage by 88.5% in 2001–2012.

The proportion of indirect obstetric death increased from 15.4% in 2001 to 62.5% in Wuhan in 2012 and exceeded the proportion of direct obstetric death in 2011. Access to emergency obstetric care or an emergency referral system can significantly reduce maternal mortality. Providers of emergency obstetric care should collaborate with other hospital departments [16]. Wuhan had four first-class medical hospitals located in the different sectors of the city and sent high-risk pregnant women to these four hospitals for treatment in accordance with the principle of treatment proximity. These actions had improved the success rate for the heart disease treatment.

This study shows that pregnancy complications is now the leading cause of maternal mortality in Wuhan and the heart disease is the most prevalent pregnancy complications. Because certain symptoms of pregnancy can have similar symptoms as heart disease, such as heart palpitations, shortness of breath, edema of the foot and ankle, patients with heart disease can be missed as a complication of pregnancy [24]. Since 2012 all pregnant women in Wuhan have been required to be screened for heart disease at their first prenatal visits. If the screening test is positive then a standard echocardiogram using an ultrasound probe will be performed. High-risk pregnancy care is provided to women with a history of pregnancy risks or current medical complications to ensure the best outcome for the mother and baby.

Improving the knowledge, attitude, and skill of health professionals can effectively reduce maternal mortality. This study showed that 74.7% of maternal deaths were avoidable and conditionally avoidable, and 50% of the avoidable and conditionally avoidable maternal deaths were due to a lack of adequate knowledge, attitude, resource and management of health care institution. Wuhan has conducted various obstetric health professional training programs and simulation exercises to improve obstetric hemorrhage treatment, and this has significantly decreased the MMR due to obstetric hemorrhage from 23.13 in 2001 to 2.66 in 2012 per 10,000 live births.

Of 91 maternal deaths, 60% resulted from a lack of adequate prenatal care which may be related to a lack of maternal health awareness, low socioeconomic status, and inaccessibility of medical services. Utilizing maternal health consultants has improved maternal health and reduced maternal mortality by providing telephone counseling, health education, medical advice and counseling on rational use of medicines, effectively urging pregnant women to enroll in prenatal care, and implementing effective health management.

Prenatal care plays an important role in reducing maternal death. In recent years, Wuhan’s government has made dramatic progress in improving the maternal health management and the quality of prenatal care, including universal prenatal registration, regular prenatal check-ups, screening and follow-up of patients with medical conditions that indicate high risk, universal hospital delivery, fetal monitoring during labor, post-natal visits, and health education promoting maternal health during pregnancy. However, since 2001 about 47.7% of the pregnant women have not received regular check-ups prior to their pregnancy and 11.6% have never received any check-ups respectively, and therefore it is still an urgent mission to provide the best quality prenatal care and to promote maternal health by providing pregnant women and new mothers with the information they need to take care of their health.

During the tenth anniversary of the Safe Motherhood Initiative, the WHO summarized key programmatic intervention priorities for reducing maternal mortality and morbidity, including improving maternal health care, reducing unsafe abortions, enhancing capacity to use maternal health services and improving emergency obstetric care [13]. Implementation of these intervention priorities should be facilitated by providing comprehensive prenatal diagnosis, treatment, management of high-risk, complicated pregnancies, and emergency obstetric care and referral system, and proving health education and information in pregnancy to help pregnant women get the care they need to have a healthy pregnancy and a healthy baby. These interventions have been successful in reducing maternal mortality in Wuhan in the past decade. It is our responsibility to share our knowledge and experiences to help other maternal health providers in China with maternal mortality reduction efforts.

## Conclusion

With the reduction in MMR in obstetric death (e.g. obstetric hemorrhage), there had been a remarkable reduction in MMR in Wuhan in 2001–2012, due to the reduction in MMR in obstetric death (e.g. obstetric hemorrhage), which may be due to (1) the improvement in the obstetric quality of perinatal care service on prevention and treatment of obstetric hemorrhage and emergency care skills, and (2) the improvement in the maternal health management and quality of prenatal care. Interventions to further reduce the MMR include several efforts such as the following: (1) designing community-based interventions, (2) providing subsidies to rural women and/hospitals for hospital delivery, (3) screening for pregnancy complications, and (4) establishing an emergency rescue system for critically ill pregnant women.
